# How Dietary Fibre, Acting via the Gut Microbiome, Lowers Blood Pressure

**DOI:** 10.1007/s11906-022-01216-2

**Published:** 2022-07-15

**Authors:** Chudan Xu, Francine Z. Marques

**Affiliations:** 1 Hypertension Research Laboratory, School of Biological Sciences, Faculty of Science, Monash University, Melbourne, Australia; 2 Heart Failure Research Laboratory, Baker Heart and Diabetes Institute, Melbourne, Australia

**Keywords:** GPR41, Fibre, Prebiotics, Metabolites, Microbiome, Immune cells

## Abstract

**Purpose of Review:**

To discuss the interplay behind how a high-fibre diet leads to lower blood pressure (BP) via the gut microbiome.

**Recent Findings:**

Compelling evidence from meta-analyses support dietary fibre prevents the development of cardiovascular disease and reduces BP. This relation is due to gut microbial metabolites, called short-chain fatty acids (SCFAs), derived from fibre fermentation. The SCFAs acetate, propionate and butyrate lower BP in independent hypertensive models. Mechanisms are diverse but still not fully understood—for example, they include G protein-coupled receptors, epigenetics, immune cells, the renin-angiotensin system and vasculature changes. Lack of dietary fibre leads to changes to the gut microbiota that drive an increase in BP. The mechanisms involved are unknown.

**Summary:**

The intricate interplay between fibre, the gut microbiota and SCFAs may represent novel therapeutic approaches for high BP. Other gut microbiota-derived metabolites, produced when fibre intake is low, may hold potential therapeutic applications. Further translational evidence is needed.

## Introduction

High blood pressure (BP), also known as hypertension, affects one in every three adults globally [1, 2]. The BP of two-thirds of hypertensive patients remains uncontrolled, especially in low- and middle-income countries [1]. According to the Global Burden of Disease study, high systolic BP is the leading risk for attributable deaths [3]. Thus, understanding the reasons why high BP remains highly prevalent and uncontrolled is crucial.A well-known risk factor for hypertension, and one of the first lines of intervention according to recent guidelines, is diet [4]. Alarmingly, in 2017, the intake of most healthy foods was suboptimal [5•]. In the same year, dietary risks were estimated to have contributed to 11 million deaths and 255 million disability-adjusted life-years (DALYs) in adults [5•]. The main cause of diet-related deaths and DALYs was cardiovascular disease (CVD) [5•]. Diet-related deaths were attributed to high sodium intake, followed by low intake of whole grains, fruits, nuts, seeds and vegetables, while DALYs were primarily attributed to low intake of whole grains [5•]. Overall, foods high in whole grains, fruits, nuts, seeds and vegetables are high in fibre. The first evidence we could identify reporting that dietary fibre lowers BP is a small clinical trial that dates from 1979 [6]. Four decades later, the evidence that overall fibre intake is associated with a lower incidence of CVD and lower BP is robust [7••, 8]. Until recently, however, we did not understand how this happened and if this was an association or indeed dietary fibre was involved in BP regulation. Since 2017, a growing body of evidence suggests this occurs via the gut microbiota, the microorganisms that inhabit the intestine [9••, 10]. In this review, we summarize the complex interplay between fibre, the gut microbiota, microbial metabolites and their molecular mechanisms, and the associated changes in BP. We review the most recent literature supporting that manipulation of the gut microbiota and/or their metabolites produced after fibre intake might be a novel therapeutic approach for hypertension.

## Dietary Fibre and Lower Incidence of CVD: the Latest Evidence

**Fig. 1:**
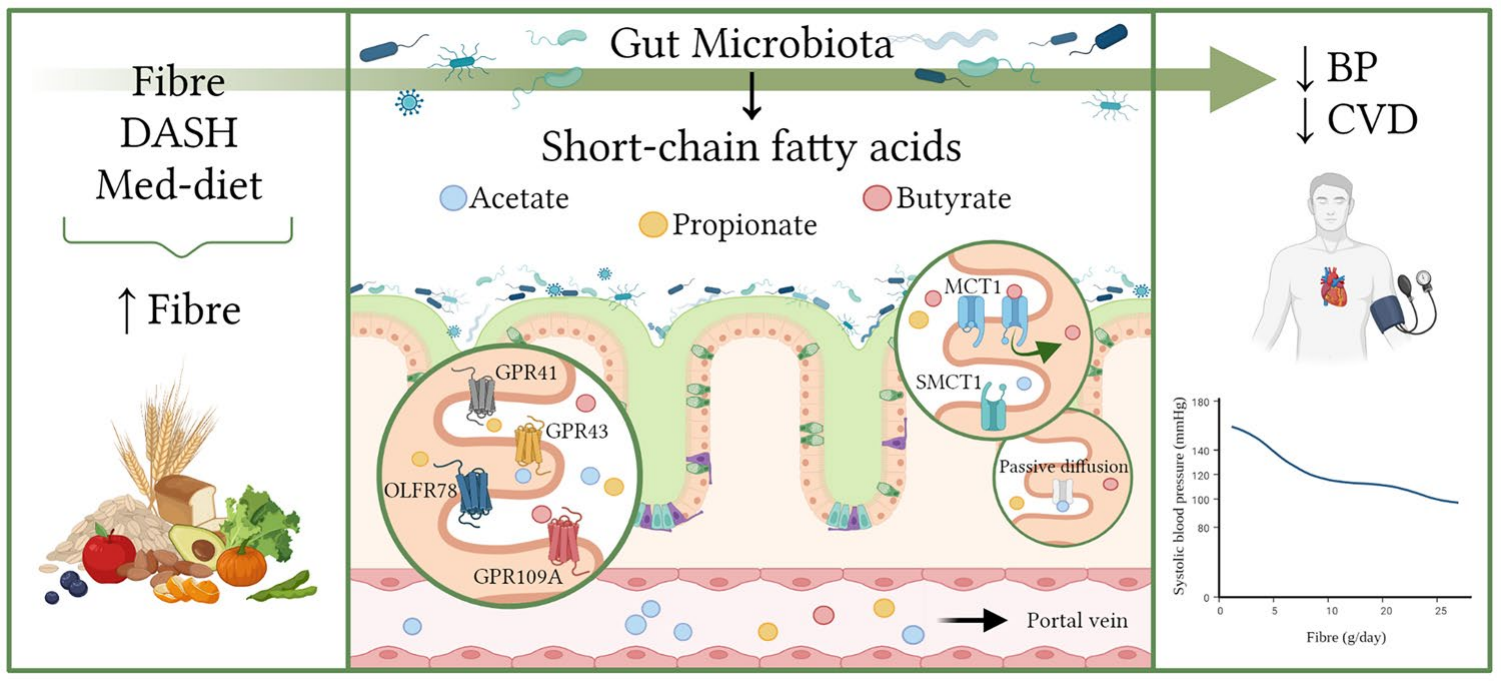
Dietary fibre, acting via the gut microbiota, lowers blood pressure. Diets high in fibre are associated with lower blood pressure (BP) and risk of cardiovascular disease (CVD). Fibres reach the colon intact, as they resist being digested or absorbed in the upper intestine. In the colon, the gut microbiota utilizes them as fuel sources and produces short-chain fatty acids (SCFAs) as by-products. These microbial metabolites have different routes to cross the intestinal epithelium: binding G protein-coupled receptors (GPCR), through transporters such as MCT1 or SMCT1, or passive diffusion. SCFAs become intracellular or available in the circulation, especially acetate, through which they communicate with distal organs and exert their effects. Legend: DASH, dietary approaches to stop hypertension; MCT1, monocarboxylate transporter 1; MED, Mediterranean; OLFR, olfactory receptor; SMCT1, sodium-coupled monocarboxylate transporter. Created with BioRender

Over the past decades, epidemiological studies and clinical trials revealed a strong association between dietary patterns and CVD (Fig. 1). A recent systematic review and meta-analysis analysed 10 randomized clinical trials (RCTs) that employed the modified Dietary Approaches to Stop Hypertension (DASH) diet, characterized by a diet low in sodium and enriched in fruits, grains, vegetables and low-fat dairy foods [11]. This showed the modified DASH diet reduced systolic BP by 3.3 mmHg and diastolic BP by 2.1 mmHg [11]. While sodium has been the focus of most studies in dietary interventions to treat hypertension, evidence supports that the DASH diet lowers BP even when sodium intake is high [12]. This reinforces the concept that improvements in BP are not only dependent on sodium [13]. Indeed, a systematic review and meta-analysis of 6 clinical trials focused on the Mediterranean diet and BP showed a small decrease in systolic (− 1.4 mmHg) and diastolic (− 0.7 mmHg) BP [14]. Furthermore, a recent RCT reported that both Mediterranean and its improved version, the Green-Mediterranean diet, significantly reduced BP [15]. Apart from the DASH and Mediterranean diets, a meta-analysis of 185 prospective studies and a total of 58 RCTs, equivalent to ~ 135 million person-years, determined that higher fibre intake reduced overall and cardiovascular mortality by 15–30%. A diet high in fibre was also associated with a lower risk of CVD [7••]. Analysis of 15 RCTs, including 1064 intervention and 988 control participants, reported that fibre reduced systolic BP by 1.27 mmHg [7••]. A more recent meta-analysis by the same authors included 12 RCTs of 878 patients with CVD or hypertension [8]. This study provided high certainty evidence showing fibre reduces systolic BP by 4.3 mmHg [8]. An additional 5 g per day of fibre was sufficient to reduce systolic and diastolic BP by 2.8 mmHg and 2.1 mmHg, respectively [8]. These are robust evidence that dietary fibre lowers BP, even without sodium interventions.

A diet rich in fibre sources has been associated with beneficial health outcomes. Dietary fibre comprises all carbohydrates that resist digestion or absorption in the small intestine and have a degree of polymerization of at least ten monomers [16•, 17•]. There are two major types of dietary fibre, non-starch polysaccharides and resistant starches (RS). Non-starch polysaccharides, the main component of plant cell walls, include soluble fibre, which is capable of dissolving in water, and insoluble fibre, which is unable to be dissolved in water [16•, 17•]. RS range from type one to five and are the energy repertory for plants and a major dietary carbohydrate source for humans [16•]. Thus, different types of fibre are diverse and their physicochemical characteristics, including solubility, viscosity and fermentability, can be variable based on different food processing methods and individual health conditions [16•]. While all types of fibre are not digested by mammalian enzymes and reach the large intestine intact, their degree of fermentation is variable. For example, certain types of soluble fibre (e.g. inulin, galactooligosaccharides, pectins) and RS are highly fermentable, while some types of insoluble fibre (e.g. cellulose and lignins present in the cell walls) have lower fermentability [16•]. Research is largely lacking on the effect of different types of fibre on BP. In particular, RS are remarkably difficult to study and quantify, as their levels vary depending on how foods are cooked and ingested. The heterogeneity of trials poses a large limitation to the direct use of these types of fibre in clinical practice. Combined with a lack of information about fibre intake in hypertensive guidelines [4], overall diets aimed to increase the intake of foods high in fibre and potassium and lower in sodium, such as the DASH or Mediterranean diets, are still the best approach—at least for now.

## Fibre Digestion by the Gut Microbiota

Fibre fermentation in the large intestine is driven by the gut microbiota [16•], the living microorganisms that inhabit the intestinal ecosystem [18•]. Thus, fibre intake not only modulates the gut microbiome, the microbiota plus their nucleic acid, but also microbial structural elements and microbial metabolites [18•, 19••]. The latest estimation suggests a ‘reference man’ has a similar number of human and bacteria cells in the body (~ 3.8 × 10^13^ each) [20]. However, a ‘reference woman’, infants and the elder were estimated to have 1.7–2.2 more bacterial than human cells in the body [20]. While the vast majority of these bacterial cells inhabit the large intestine [20], the number of other microorganisms (e.g. viruses, fungi) remains unaccounted for.

Two recent crossover trials investigated the effect of two purified fibres, arabinoxylan and inulin; a mixture of five types of fibre; and RS on the microbiota [19••, 21]. These studies independently identified that each type of fibre was associated with distinct microbial responses [19••, 21]. Likewise, small chemical structural changes in type 4 (chemically modified) RS drove different effects on the gut microbiota and production of their metabolites in humans [22•]. However, a high inter-individual response is regularly observed in such interventions, highlighting the need for a precision approach to nutrition and microbiome interventions, as well as a better understanding of the individual baseline microbiome [23].

The microbiota inhabits the gut and gut mucosal barrier, and supports the maintenance of a healthy gut epithelial barrier via metabolite production, further discussed below [24]. This physical barrier prevents pathogenic colonization and invasion. In fibre-rich diets, there is a proliferation of gut microbiota that digests fibre, supporting the maintenance of the gut epithelial barrier [25]. In fibre-free diets, there is a shift in the gut microbiota composition, leading to the proliferation of bacteria that digest the intestinal mucus layer instead [25]. This contributes to the breakdown of the gut epithelial barrier; the entrance of undesirable microbes and their substances into the host’s systemic circulation; and the subsequent activation of a chronic inflammatory state [25]. A similar chronic inflammatory state is observed in CVD and high BP [26•]—this observation suggests gut dysbiosis and breakdown of the gut epithelial barrier may be involved in the development of these diseases.

## Short-Chain Fatty Acids: the Microbial Products Derived from Fibre Fermentation

In the large intestine, dietary fibre fermentation by the gut microbiota leads to the generation of SCFAs as by-products (Fig. 1) [27•]. Several bacteria are involved in this process via distinct biochemical pathways, summarized in Table 1. However, we still do not completely understand the enzymatic machinery necessary to degrade certain types of fibre, such as RS [23]. The three major SCFAs derived from microbial metabolism are acetate, propionate and butyrate, previously reported to be in a ratio of approximately 60:20:20 in the colon of sudden death victims [28]. We analysed faecal levels of SCFAs in a multi-site cohort study and found that acetate corresponded to 55% of total SCFAs, while propionate and butyrate were 17% each, with the remaining 11% being accounted for iso-butyric, iso-valeric, valeric and caproic acids [29••]. SCFAs have 1–6 carbon-based anions, with acetate having 2, propionate 3 and butyrate 4 carbons [27•].

**Table 1:** Biosynthesis pathways and gut bacteria are involved in generating the three main short-chain fatty acids (acetate, butyrate and propionate), and the main host receptors that sense these metabolites

**Metabolite (number of carbons)**	Possible microbial biochemical pathways*	SCFA-producing bacteria*	**Receptors and their expression in host cells**
Acetate (C2)	Classical pathway: Pyruvate acetyl-CoA acetate [86]	▪ *Prevotella* spp. ▪ *Ruminococcus* spp. ▪ *Bifidobacterium* spp. ▪ *Bacteroides* spp. ▪ *Clostridium* spp. ▪ *Streptococcus* spp. ▪ *Akkermansia muciniphila* ▪ *B. hydrogenotrophica* [87]	▪ GPR41 (*FFAR3*)—expressed in pancreatic cells, neurons and white adipocytes (host metabolism) [88–90] ▪ GPR43 (*FFAR2*)—expressed in enterocytes and immune cells [32, 91, 92] ▪ OLFR78 (*OR51E2*)—expressed in renal afferent arterioles and extra-renal vascular beds [52•]
	Wood-Ljungdahl pathway [93]	▪ *Clostridium* spp. ▪ *Streptococcus* spp. [93]	
Propionate (C3)	Acrylate pathway [94]	▪ *Coprococcus catus* [94] ▪ *Megasphaera* spp. [95]	▪ GPR41 (*FFAR3*) ▪ GPR43 (*FFAR2*) ▪ OLFR78 (*OR51E2*) [52•]
	Succinate pathway [94]	▪ Bacteroidetes [96] ▪ *Veillonella* spp. [95]	
	Propanediol pathway [94]	▪ *Akkermansia municiphilla *[97] ▪ *Roseburia inulinivorans* [98] ▪ *Salmonella enterica* serovar Typhimurium [99] ▪ *Ruminococcus obeum *[95]	
Butyrate (C4)	Classical pathway: Butyrate kinase (two acetyl-CoA molecules butyryl-CoA Butyrate) [95]	▪ *Coprococcus comes* ▪ *Coprococcus eutactus *[95]	▪ GPR41 (*FFAR3*) ▪ GPR43 (*FFAR2*) ▪ GPR109A (*HCAR2*)—adipocytes, gut epithelium and immune cells [100, 101]
	Butyryl-CoA: acetate-CoA-transferase pathway [95]	▪ *Ruminococcus bromii* [102] ▪ *Faecalibacterium prausnitzii* [103] ▪ *Eubacterium rectale* ▪ *Eubacterium hallii* [103] ▪ *Roseburia* spp. ▪ *Anaerostipes* spp. ▪ *Coprococcus catus* [95]	

^*^Not limited to the list. *BP*, blood pressure; *FFAR*, free fatty acid receptor; *GPR*, G protein-coupled receptors; *HCAR2*, hydroxycarboxylic acid receptor 2

Although SCFAs can be ingested or produced by other metabolic processes, bacterial fermentation of fibre is the major source of SCFA production in the human body [27•]. Different types of fibre are also fermented in distinct regions of the colon [17•]. Rapidly fermented fibres, such as inulin, are fermented in the proximal region, while moderate- and slow-fermented fibres, such as RS type 2, are fermented in the proximal and transverse regions [17•]. This means that levels of SCFAs vary along the colon, with distal regions having lower levels due to the depletion of fermentable fibres, leading to protein fermentation instead [17•]. This is also reflected in changes in pH in the different intestinal regions, with the proximal region having the lowest and the distal region having the highest pH [17•]. While SCFAs were measured in faecal and blood samples in most of the human studies, these may not reflect the levels produced inside the intestine, particularly, in different colonic regions.

SCFAs, in particular butyrate, are absorbed by intestinal epithelial cells by the monocarboxylate transporter 1 (MCT1, encoded by the gene *SLC16A1*) and sodium-coupled monocarboxylate transporter (SMCT1, gene *SLC5A8*), promoting cellular metabolism [27•]. Butyrate, as a major source of ATP for colonocytes, leads to the maintenance of the gut epithelial barrier [27•]. It also depletes intracellular oxygen which leads to the stabilization of the transcription factor hypoxia-inducible factor 1 (HIF1), which coordinates the expression of tight junction genes in the intestinal epithelial barrier [30]. Although all SCFAs inhibit histone deacetylases (HDACs), butyrate is the most potent [27•]. Moreover, SCFAs act via signalling cascades when they bind to the G protein-coupled receptors (GPCRs)—GPR41, GPR43 and GPR109A (Table 1) [27•]. These receptors are mostly expressed on the surface of immune and gut epithelial cells [31]. Their function in hypertension is further discussed below.

The majority of SCFAs diffuse through the intestinal epithelium to the lamina propria, entering the circulation via the portal vein [27•]. SCFAs can be utilized by different cell types, including enteroendocrine L-cells, beta cells in the pancreas and immune cells [32, 33]. While propionate is preferentially metabolized by hepatocytes, acetate is the only SCFA that is usually detected at physiological concentrations in the host’s systemic circulation [27•]. In our studies, acetate was the main SCFA detected in plasma (94%), while propionate and butyrate corresponded to ~ 3% each [29••]—reflecting that only a minority of these SCFAs become systemically available. However, acetate can act as a substrate and be converted into fellow SCFAs [27•]. Nevertheless, the amount of SCFAs in the circulation and their turnover rate are also tightly regulated by the endogenous energy level, such as glucose, fatty acids and ketone bodies [34].

## SCFAs Mediate Downstream Effects Outside the Intestine

**Fig. 2:**
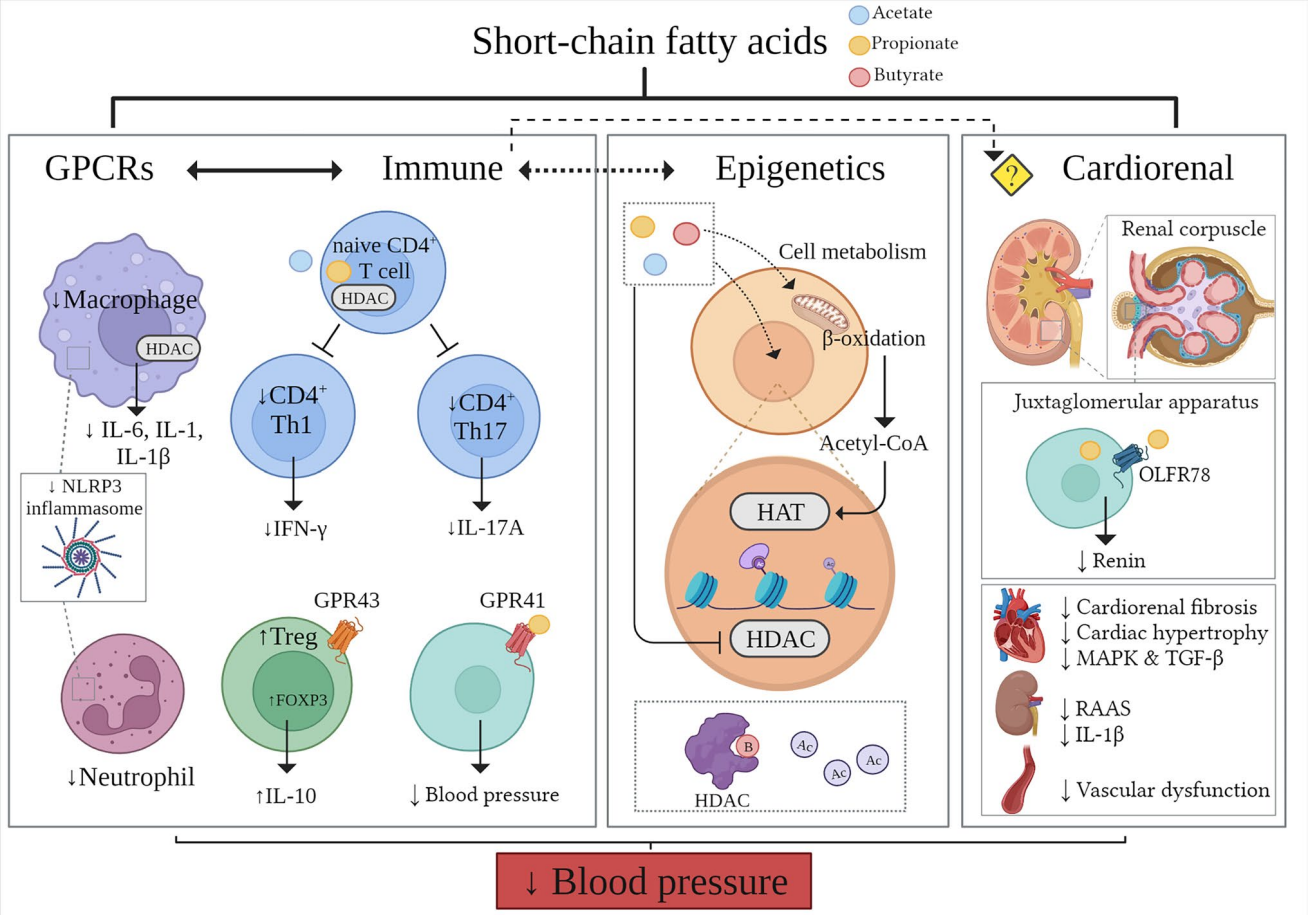
Known molecular mechanisms of action of short-chain fatty acids and how they may lower blood pressure. The three main short-chain fatty acids (SCFAs), acetate, propionate and butyrate, have multi-faceted actions via G protein-coupled receptors (GPCR), epigenetic, immune-dependent and immune-independent mechanisms that together may lower blood pressure and elicit a cardiorenal protective effect. Legend: Ac, acetyl group; GPCRs/GPR, G protein-coupled receptors; HAT, histone acetyltransferase; HDAC, histone deacetylases; IL, interleukin; IFN, interferon; MAPK, mitogen-activated protein kinases; NLRP3, NOD-, LRR- and pyrin domain-containing protein 3; OLFR, olfactory receptor; RAAS, renin-aldosterone-angiotensin system; TGF, transformation of growth factor; Th, helper T; T_reg_, regulatory T. Created with BioRender

It is estimated that 60% of colonic SCFAs diffuse from the lumen to the lamina propria with the remaining portion taken up directly by MCT1 and SMCT1 transporters in the epithelial cells [35]. As mentioned, SCFAs can bind to GPR41, GPR43 and GPR109A expressed on diverse cell types, including gut epithelial cells, adipocytes, enteroendocrine L-cells, innate immune cells and neurons [36, 37•]. Intracellular SCFAs can regulate epigenetic genes by HDAC inhibition [38], where butyrate may act as a competitive inhibitor and might occupy the hydrophobic binding cleft of the active site [39]. Moreover, mainly in the liver, intracellular SCFAs are essential substrates for β-oxidation and the Krebs cycle. A study investigated the roles of SCFAs in cell metabolism, in which mice were infused with physiological quantities of isotope labelled SCFAs into the cecum [34]. It identified butyrate as the main substrate for lipogenesis, propionate for gluconeogenesis and a minor proportion of acetate and butyrate for cholesterol synthesis [34]. At the epigenetic level, acetyl-CoA derived from β-oxidation, glycolysis and lipid metabolism can modulate histone acetyltransferase, the antagonistic enzyme of HDAC, activity in the nucleus [40]. The several downstream mechanisms involved in the actions of SCFAs that may impact BP are summarized in Fig. 2.

SCFAs and other gut microbiota-derived metabolites are key in microbiota-host communication as they can modulate distal organ physiological and molecular functions. Indeed, using in vivo carbon-11 acetate and positron emission tomography, i.v. and colonic acetate were mostly absorbed by the brain, heart and liver [41]. Moreover, transcriptomic analyses of 3-week administration of a high-RS diet and acetate in the drinking water showed downregulation of the renin-aldosterone-angiotensin system (RAAS) and interleukin (IL)-1β in the kidney, and downregulation of mitogen-activated protein kinases (MAPK) and transformation of growth factor β (TGFβ) signalling in the heart, providing evidence for a gut-cardiorenal communication [9••]. Intervention with high RS and acetate increased the mRNA and protein levels of renal angiotensin-converting enzyme 2 (ACE2) via GPR41/43/109A signalling [42]. Recent evidence showed even maternal dietary fibre modulated the molecular and cellular composition of the adult offspring’s heart [43]. These demonstrate compelling evidence that SCFAs have important roles outside the intestine that may impact BP and CVD.

## SCFAs and BP in Experimental Hypertension

Gut dysbiosis is characterized by changes to the structure of the gut microbiota and a compromised gut epithelial barrier. An important component of hypertensive states may be changes in the capacity of the microbiota to produce SCFAs, which may lead to the breakdown of the gut epithelial barrier. Indeed, lower SCFA-producing bacteria and increased intestinal permeability were reported in both hypertensive models (angiotensin II, DOCA/salt mice and spontaneously hypertensive rats (SHR)) and human hypertensive patients [9••, 29••, 44••, 45••, 46, 47]. Early studies using acute administration of SCFAs suggested these metabolites may have a BP-lowering effect: SCFAs caused vasodilation in dogs [48, 49], rat caudal arteries [50] and human colonic arteries from 6 donors [51]. More recently, acute delivery of propionate resulted in a dose-dependent reduction in BP via GPR41 signalling [52•]. Furthermore, acute administration of acetate reduced heart rate and mean arterial pressure—the use of atenolol to block sympathetic tone abolished the effect on heart rate, but the BP-lowering effect persisted [53].

The long-term effects of SCFAs have only been determined more recently, with a growing number of studies demonstrating the three main SCFAs were able to reduce BP and improve cardiac performance in independent studies (Table 1). Similarly, to a high-RS diet, we reported that magnesium acetate supplementation in the drinking water reduced BP and cardiorenal fibrosis in the DOCA-salt model [9••]. This was followed by further validations of a BP- and fibrotic-lowering effect of magnesium acetate, sodium propionate and sodium butyrate, as well as a combination of all three in the Ang II model, even in combination with a low-fibre diet [54••]. Acetate led to a decrease in the calculated total peripheral resistance and sodium to potassium excretion, but no changes were observed in cardiac output, stroke volume or plasma noradrenaline [54••]. BP-lowering effect induced by SCFAs has been independently validated by others: butyrate supplementation in Ang II mice reduced their BP [55], and propionate supplementation in Ang II-infused apolipoprotein E knockout (*Apoe*^*−/−*^) mice ameliorated cardiac hypertrophy, fibrosis and vascular dysfunction [56••]. Unpublished data from our team has compared the effect of magnesium and sodium acetate, which determined that the magnesium version had a larger BP-lowering effect than the sodium one. Unfortunately, butyrate and propionate are usually only available in sodium forms. This represents a barrier to their direct clinical use.

Consistently, butyrate intervention was shown to reduce BP in both hypertensive (SHR [57], Ang II-infused Sprague Dawley rats [58]) and normotensive (Wistar Kyoto [59]) rats. Sodium butyrate decreased the level of an endotoxin, lipopolysaccharide (LPS), in the plasma and associated expression of genes for the interleukin *Il1β* [57], the inflammasome-component *Nlrp3*, and the chemokine *Mcp1* in cardiac tissue via COX2/PGE2 pathway inhibition [58]. In another relevant study, *Apoe*^*−/−*^ mice fed with a high-fat diet as a model of atherosclerosis, treatment with propionate reduced intestinal cholesterol and blood low-density lipoprotein (LDL) levels that ameliorated the disease phenotype [60]. The molecular mechanisms of SCFAs identified so far are discussed below.

Olfactory receptor 78 (OLFR78, encoded by the gene *Or51e2*) is another GPCR that responds to SCFAs, particularly acetate and propionate [52•]. OLFR78 is expressed in the vascular smooth muscle and renal juxtaglomerular apparatus, where it was detected to modulate renin secretion [52•]. An acute propionate (10 mM) administration was assessed in *Olfr78*^*−/−*^ mice. Due to the lack of OLFR78, the renin response was abolished and, thus, an acute drop in BP was observed, confirming that OLFR78 raised BP and antagonized the hypotensive effects of propionate [52•]. In a recent study, OLFR78 was investigated in chronic BP regulation, showing that *Olfr78*^*−/−*^ mice had lower renin levels but no differences in baseline BP compared to their WT counterparts [61].

Furthermore, evidence supports that propionate has a hypotensive effect via GPR41. Acute propionate administration caused a minimal reduction in BP response in *Gpr41*^±^ heterozygotes and a modest increased BP response in *Gpr41*^*−/−*^ animals [52•]. This demonstrated that, with the lack of GPR41, there is a reduction in the number of receptors for propionate and, thus, their signalling that impacts BP responses. In addition, *Gpr41*^*−/−*^ mice were reported to have higher systolic hypertension compared to WT animals [62•]. When comparing 3-month versus 6-month old *Gpr41*^*−/−*^ mice, the older group was found with elevated pulse wave velocity, but no increase in ex vivo aorta stiffness, suggesting that endothelial GPR41 lowers baseline BP by decreasing the vascular contractile activity without altering vascular characteristics [62•]. Moreover, one study compared the phenotype of naïve single GPR41, GPR43, GPR109A knockout and GPR43/109A double knockout mice [54••]. At 10 weeks of age, these animals showed no changes in BP, but all presented differences in cardiac function and fibrosis [54••]. Interestingly, the GPR43/109A double knockout mice had a more severe phenotype than individual GPCR knockouts [54••]. Hence, the role of SCFAs-sensing receptors seems intricate—since these receptors act on similar pathways [37•], deletion of only one or two receptors might trigger compensatory mechanisms via the other(s). More comprehensive studies assessing the function of these receptors as well as MCT1 and SMCT1 in hypertension are needed.

## SCFAs and BP in Essential Hypertension

A non-placebo controlled RCT showed healthy participants with 20-g supplementation of dietary fibre, inulin, for 6 weeks had a significant increase in serum butyrate and reduced systolic (− 6.3 mmHg) and diastolic (− 3.1 mmHg) BP [63•]. Levels of pro-inflammatory cytokines IL-4, IL-8 and TNFα were also reduced [63•]. This provides some translational evidence that SCFAs may lower BP in essential hypertension. However, clinical studies that assessed the levels of SCFAs in hypertensive patients have had inconsistent results (summarized in Table 2). On the one hand, untreated hypertensive patients, diagnosed by ambulatory BP monitoring, had higher plasma acetate and butyrate that positively correlated with systolic and diastolic BP [29••]. The bacterial pathway acetate-CoA ligase (ADP-forming), which converts ATP, CoA and acetate into ADP, acetyl-CoA and phosphate, was also upregulated in essential hypertension [29••]. BP variability, measured as morning BP surge, was negatively associated with total plasma SCFAs and, in particular, acetate [64]. Similarly, a higher level of circulating butyrate was found positively associated with ambulatory arterial stiffness index, a critical indicator of arterial function in cardiovascular diseases [65]. A possible explanation is that the sensing and uptake of SCFAs from the circulation into relevant cells are defective. This could be explained by observed reduced levels of *GPR43* mRNA in hypertensive patients, and the negative association between both *GPR41* and *GPR43* mRNA and arterial stiffness [29••, 65]. On the other hand, acetate and butyrate levels were lower in plasma from hypertensive patients, both untreated and patients taking anti-hypertensive drugs [66, 67•]. Furthermore, hypertensive subjects had a higher level of acetate, butyrate and propionate in their stool samples [66, 68]. The detection of SCFAs in the faecal samples might indicate that their absorption efficacy in hypertension has been decreased as less than 5% of these metabolites are expected to be excreted in faeces. Further studies in larger cohorts with well-characterized BP are needed to clarify the direction of the association between SCFAs and essential hypertension.

**Table 2:** Cross-sectional clinical studies that assessed the levels of the three main short-chain fatty acids (acetate, butyrate and propionate) in hypertension

**Blood pressure measurement**	**Groups, sample size**	**Gender and mean age (y)**	**Main findings in hypertension**	**Ref**
Ambulatory BP monitoring	Untreated HTN (*n* = 23) vs NT (*n* = 47)	Men and women HTN (60.3 ± 6.6) NT (59.2 ± 7.7)	↑ plasma acetate and butyrate, positively correlated with SBP and DBP No change in faecal SCFAs ↓ levels of GPR43 expression in immune cells	[29••]
Office BP	Untreated HTN (*n* = 29) vs NT (*n* = 32)	Men and women HTN (53.7 ± 9.6) NT (41.1 ± 9.1)	↓ plasma acetate & butyrate ↑ faecal acetate, propionate, butyrate	[66]
Office BP	HTN (*n* = 22) vs NT (*n* = 18)	Men and women (age not reported)	↓ plasma butyrate	[67•]
Ambulatory BP monitoring	HTN (*n* = 38); borderline (*n* = 7); NT (*n* = 9)	Men HTN (46.2 ± 11.4); borderline HTN (50.3 ± 13.3); NT (52.5 ± 8.2)	No change in serum and urine SCFAs ↑ faecal acetate, propionate and butyrate in HTN	[68]

*DBP* diastolic blood pressure, *HTN* hypertensive patients, *NT* normotensive participants, *SBP* systolic blood pressure, *SCFAs* short-chain fatty acids

## The Effects of SCFAs on a Broad Range of Immune Cells Important for Hypertension

SCFAs have anti-inflammatory effects on several immune cells [27•], which are also associated with the development of hypertension [26•]. Cytokines such as IL-17 and IFN-γ were reported to promote the development of hypertension, whereas IL-10 attenuated the disease [26•]. The direct link between the anti-inflammatory actions of SCFAs in lowering BP is still missing. In patients with ulcerative colitis, butyrate decreased the number of macrophages and neutrophils in the plasma and intestinal lamina propria via inhibition of NF-KB nuclear translocation [69]. Lower levels of pro-inflammatory cytokines IL-6 and IL-12 were identified in intestinal macrophages and bone marrow-derived macrophages treated with butyrate via an HDAC-dependent mechanism [70]. Similarly, through HDAC inhibition, propionate and acetate increased acetylation of the mTOR pathway that blocks T helper 17 (Th17) and T helper type 1 (Th1) differentiation [69]. As a result, these cells secrete fewer cytokines, including IL-17, interferon (IFN)-γ and IL-10 [71].

SCFAs may also have a direct anti-inflammatory role via differentiation of naïve T cells into regulatory T cells (T_regs_), increasing *Foxp3* expression via GPR43 [72]. In mice, a 3-week intervention with RS or acetate increased the number of T_regs_ and upregulated methylation of genes associated with T_regs_ function in splenocytes [54••]. A group of SCFA-producing strains of Clostridia isolated from a healthy human faecal sample, enriched in T_regs_-inducing species, was transferred into germ-free mice. This cluster of bacteria generated a TGF-β-rich environment which favoured the differentiation of colonic T_regs_ [73]. In humans, however, a short (5 days) intervention that increased the systemic levels of acetate and propionate did not change the levels of T_regs_ [74]. This suggests issues with the translation or that longer term interventions may be needed in humans.

Overall, these studies showed that SCFAs have a direct effect on a broad range of immune cells, which in turn may either promote or attenuate hypertension. It remains unclear why SCFAs have different preferences for receptor activation and/or HDAC inhibition within different cell types.

## What Happens to BP when Fibre Intake Is Low

Back in 1979, a study demonstrated participants with a low-fibre intake diet had higher systolic and diastolic BP [6]. In the same study, 11 participants, who routinely were on a high-fibre diet, decreased their total dietary fibre intake by 55% for 4 weeks, resulting in an increase in their mean systolic and diastolic BP by 6.8% and 3.8%, respectively [6]. Now that we understand the importance of the gut microbiota for fibre fermentation and that the gut microbiota changes rapidly, it is important to differentiate association from causation in the change in BP. Germ-free mice, which do not possess any microbiota, are a very powerful tool to address this question [75]. Faecal microbiota transplantation (FMT) from low- and high-fibre fed mice into germ-free animals demonstrated that a low-fibre diet is not merely associated with a higher incidence of high BP [54••]. The gut microbiota resulting from long-term low-fibre intake triggered and promoted the genesis of higher systolic (+ 17 mmHg) and diastolic (+ 14 mmHg) BP and cardiorenal hypertrophy in mice, showing that this microbiota is hypertensinogenic [54••]. By supplementing acetate, propionate and/or butyrate in the water of conventional Ang II mice fed with a low-fibre diet, this hypertensinogenic effect was ameliorated [54••].

Furthermore, patients with an advanced stage of chronic kidney disease (CKD) with a low-fibre intake (< 25 g/day) had a lower estimated glomerular filtration rate and a higher level of C-reactive protein, IL-6 and the uremic toxin indoxyl sulphate, indicating reduced renal function and increased inflammatory markers [76]. Similarly, in children with CKD, an inverse association was observed between fibre consumption and serum concentration of protein-bound uraemic toxins, such as indoxyl sulphate, p-Cresol sulphate, p-Cresol glucuronide and indole acetic acid [77]. This correlation was dose-dependent: for every gram/day increase in fibre consumption, there was a small decrease in particular metabolites, which ameliorated their accumulation in the kidney [77].

Therefore, a diet lacking sufficient fibre may play a role in hypertension and CVD pathogenesis. A possible explanation is a deficiency in fibre fermentation and, thus, SCFAs in the proximal and transverse colon, resulting in lower anti-inflammatory effects in the interstitial epithelial cells and the systemic circulation. In return, increased protein fermentation takes place earlier in the colon, which might lead to exposure of the mucosal layer to potential harmful metabolites, such as phenols and hydrogen sulphide [17•]. However, the specific processes that happen inside the intestine and over-flow to the systemic circulation when fibre intake is low are yet to be determined.

## Challenges in the Field

There has been an over-reliance on the abundance of microorganisms instead of their function. As the pathogenesis of hypertension is a complex interplay between several systemic systems, a similar approach regarding the microbiome needs to be considered in this setting. It is ideal to integrate multi-omics studies, such as metagenomics, metatranscriptomics, metaproteomics and metabolomics, which will provide a more comprehensive understanding of BP regulation from a microbiome perspective. There is evidence that SCFA producers such as *Ruminococcus* spp. are less prevalent in essential hypertension, and that there is a significant shift in the gene pathways of the human hypertensive microbiome [29••]. However, metatranscriptomic or metaproteomic studies, showing a shift in the expression and function of microbial SCFA-producing genes to determine a cause-effect relationship, for example, are still absent in hypertension. Sometimes, in vitro models cannot recapitulate in vivo, especially when assessing complex microbial ecosystems such as the one found in the human intestine [17•]. This complexity can be demonstrated by the findings of an RCT aimed at reducing sodium which resulted in an increase in the levels of plasma butyrate in women [78].

In the last decade, we have seen an expansion of studies investigating gut microbiota-derived metabolites other than SCFAs in CVD—an example being trimethylamine N-oxide (TMAO) [79]. By combining convention and germ-free animals, a study identified four upregulated but under-studied metabolites in plasma samples of conventional Ang II mice [80]. This included 4-ethylphenyl sulphate and p-Cresol sulphate, with another eight metabolites downregulated [80]. In faecal samples, 25 metabolites, including choline phosphate and taurohyodeoxycholic acid, were upregulated, while 71 were downregulated [80]. Additionally, β-hydroxybutyrate, a metabolite derived from the liver, was decreased in the circulation with a high-salt diet in hypertensive rats [81]. This downregulation was associated with increased activation of the inflammasome, which in turn increased the risk of hypertension [81]. There are several challenges in the identification of novel metabolites and their roles, as metabolomics tools are still considered emerging. These include a lack of validation of some putative metabolites or tools for absolute quantification, a large array of synonymous names for the same metabolites and a requirement to use different analysis tools for different metabolites (e.g. SCFAs vs other metabolites), amongst others [82].

## Leveraging Fibre as Future Therapeutic Approaches for Hypertension

Lifestyle changes remain one of the first lines of intervention in hypertension [4]; however, they fail to promote an increase in the quantity and quality of fibre. Guidelines on the use of prebiotic foods, which selectively stimulate the growth of health-promoting bacteria, are needed. These foods include, for example, highly fermentable fibre such as inulin, sugar gum and pectin. Future interventions involve designing and developing probiotics (i.e. live bacteria) that assist in fibre digestion and SCFA production. This will also require individuals to sustain a fibre-rich diet as a food supply for the microbes to survive and populate the gut. Finally, there is also an opportunity for direct administration of SCFAs as a postbiotic therapy. One RCT aimed at assessing the direct effect of the SCFAs acetate and butyrate to lower BP in human hypertension is in progress [83]. Nevertheless, this might not be a suitable approach for all patients if patients have lower expression of GPR41 or GPR43, making them less responsive to SCFAs.

Other potential approach includes FMTs from healthy donors with enriched SCFA-producing bacteria or T_regs_-inducing bacteria. An RCT on the potential of FMTs to lower BP has been described [84], but the results are yet to be available. Moreover, interkingdom interactions within the gut could be leveraged: bacteriophages could be used to target and kill specific bacteria that produce detrimental metabolites from a low-fibre diet. Another approach could include the development of inhibitors for bacterial genes that produce detrimental metabolites, once these are identified, such as the one developed for TMAO’s precursor [85]. Nonetheless, all the above should be adjunctive therapies that complement other types of treatment or management, and it will require extensive RCTs to confirm these promising therapies.

## Conclusions

Evidence from the last four decades supports that dietary fibre lowers BP and decreases cardiovascular and all-cause mortality. The mechanisms involved have only become evident recently, supporting the gut microbiota has a key role in this process via the production of SCFAs. These metabolites have multi-faceted actions via GPCRs, epigenetic, immune-dependent and immune-independent mechanisms that together may elicit changes to BP and cardiorenal function. Alternatively, a lack of dietary fibre fosters a gut microbiota that also seems detrimental to cardiovascular health, leading to higher BP. The specific metabolites and mechanisms driving this are, however, unknown. Translational evidence for the direct use of SCFAs to lower BP in hypertensive patients is warranted, together with identification and selective inhibition of the production of detrimental metabolites associated with low-fibre intake.
